# *IGF1* gene is epigenetically activated in preterm infants with intrauterine growth restriction

**DOI:** 10.1186/s13148-020-00901-w

**Published:** 2020-07-16

**Authors:** Masato Kantake, Naho Ikeda, Hirofumi Nakaoka, Natsuki Ohkawa, Toshitaka Tanaka, Kazuki Miyabayashi, Hiromichi Shoji, Toshiaki Shimizu

**Affiliations:** 1 Neonatal Medical Center, Juntendo University Shizuoka Hospital, 1192 Nagaoka, Izunokuni, Shizuoka 410-2295 Japan; 2 Human Genetics Laboratory, Department of Genomics and Evolutionary Biology, National Institute of Genetics, 1111 Yata, Mishima, Shizuoka 411-8540 Japan; 3 Department of Cancer Genome Research, Sasaki Institute, Sasaki Foundation, 2-2 Kandasurugadai, Chiyoda-ku, Tokyo, 101-0062 Japan; 4 Perinatal Medical Center, Juntendo University Shizuoka Hospital, 1192 Nagaoka, Izunokuni, Shizuoka 410-2295 Japan; 5 Department of Pediatrics and Adolescent Medicine, Juntendo University, 3-1-3 Hongo, Bunkyo-ku, Tokyo, 113-8431 Japan

**Keywords:** IGF1, P2 promoter, Methylation, Epigenetics, Intrauterine growth restriction, IUGR, Preterm, Newborn infant

## Abstract

**Background:**

IGF1 is a key molecule in the regulation of growth and metabolism. Low IGF1 secretion is known to cause growth restriction in childhood, as well as deregulated lipid metabolism, cardiovascular disease, and diabetes in adulthood. The *IGF1* gene P2 promoter is highly methylated, resulting in low secretion of IGF1 in small infants and children. However, it is unknown when this methylation occurs. The aim of study was to clarify the point when this epigenetic program occurs during intrauterine development.

We analyzed 56 preterm infants born before 32 weeks of gestation, including 19 intrauterine growth restriction (IUGR) infants whose birth weights were lower than − 2SD calculated by the Japanese datasets. We extracted genomic DNA from whole blood at birth; methylation of the six CpG sites in the *IGF1* P2 promoter was analyzed by the bisulfite amplicon method using the MiSeq platform.

**Results:**

In contrast to term infants and children, the methylation of all six CpG sites positively correlated with body weight and body length at birth. *IGF1* P2 promoter methylation levels were significantly reduced in all six CpG sites in infants with IUGR.

**Conclusions:**

These findings indicated that the *IGF1* gene is epigenetically activated before 32 weeks of gestation in infants with IUGR and that the activated gene may become suppressed after this time point. This study may provide new insights to prevent the onset of adult diseases and to aid in nutritional management for preterm birth infants in neonatal intensive care units.

## Background

The early-life environment has long-term effects on health and development in later life. According to the Developmental Origins of Health and Diseases hypothesis, when the antenatal environmental conditions are adverse for a growing fetus, the fetus adapts to this environment to survive in utero and ex utero life [1]. These adaptations include reduced production of and sensitivity to IGF1. IGF1 is a mitogenic hormone that is produced by most organs (mainly by the liver), which stimulates systemic body growth [2–4]. Animal studies and analysis of genetic defects in humans have reported IGF1 expression in placental and fetal tissues and identified an essential role for IGF1 in fetal growth [3–5]. Serum IGF1 is also known to regulate postnatal growth in childhood [6–8]. In addition, associations between low IGF1 and deregulated lipid metabolism, cardiovascular disease, diabetes, and altered metabolic profile of diabetic patients in adulthood have been reported [9].

IGF1 is detectable in many fetal tissues from the first trimester, suggesting that it plays a role in early fetal development. However, increased levels of circulating IGF1 during the period of fetal life that corresponds to the third trimester, especially after 32 weeks of gestation, suggest that IGF1 is more important in the later months of fetal growth [10].

Although parental height correlates with offspring height [11], research on the association between the *IGF1* gene and human stature indicated that associated genetic factors, such as allelic polymorphism and DNA mutations, only explained a minor part of the expected heritable fraction [12]. On the other hand, epigenetic modifications, such as DNA methylation, can contribute to alteration of gene expression in a heritable manner without affecting the underlying genomic sequences. This hypothesis may explain instances of heritable adaptations to changing environmental conditions, such as human stature [13].

There are several transcripts of the *IGF1* gene from different transcriptional start sites. Class 1 transcripts have initiation sites on exon 1 and are driven by the P1 promoter, while class 2 transcripts use exon 2 as a leader exon and are driven by the P2 promoter [14–16]. *IGF1* transcripts initiating at P1 are constitutively expressed in many tissues, while transcripts initiating at P2 are expressed primarily, but not exclusively, in the liver [17].

Ouni et al. examined whether CG methylation of the two promoters (P1 and P2) of the *IGF1* gene is a potential epigenetic contributor to the individual variation in circulating IGF1 and stature in growing children. Their observations introduced epigenetics as an individual determinant of child growth and serum IGF1 level. Six CpG sites, CG108, CG137, CG207, CG218, CG224, and CG232 in the P2 promoter of the *IGF1* gene, especially CG137, are the first epigenetic quantitative trait loci reported in humans [17]. The methylation of these six sites is associated with growth hormone responsiveness of children presenting with short stature at 8–9 years of age [18]. Moreover, Stunff et al. reported that CG137 methylation is associated with birth length in term newborn infants [19].

To determine whether individual variation in CpG site methylation is determined in the postimplantation embryo at the time of primary shaping of the methylome or as a result of maternal signals transmitted through the placenta at a postembryonic stage of intrauterine life, we investigated the methylation status of the *IGF1* P2 promoter including CG137 in preterm (< 32 weeks of gestation) infants with and without intrauterine growth restriction (IUGR).

## Methods

### Participants

This study was approved by the Juntendo University Ethics Committee and conducted in accordance with the principles of the Declaration of Helsinki. We analyzed 56 preterm infants born before 32 weeks of gestation, including 19 infants with IUGR whose birth weights were lower than − 2SD calculated by the Japanese datasets (http://jpse.umin.jp/taikakubirthlongcrossv1.xlsx); all were admitted to the neonatal intensive care unit (NICU) of Juntendo University Shizuoka Hospital between July 2017 and August 2019, and their parents had provided written informed consent for inclusion in the study.

### Sample collection

A sample of 10 μL of whole blood was taken and frozen immediately at the time of admission to the NICU as part of routine blood sampling and stored at − 80 °C until analysis. Genomic DNA was extracted from the samples in August 2019.

### Methylation analysis

**Fig. 1:**
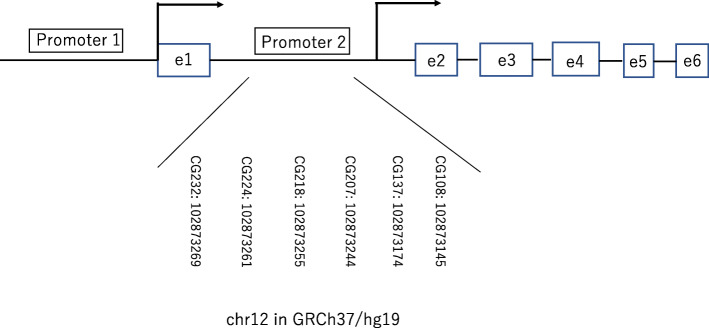
The *IGF1* gene promoters (P1 and P2). Broken arrows show the transcription start site (TSS) of each promoter. The six analyzed CG sites are shown with their locations on the chromosome. CGs are denominated according to their distance from the P2 promoter major TSS

Genomic DNA was extracted from 10 μL of frozen whole blood using a DNeasy Blood & Tissue Kit (Qiagen, Hilden, Germany); bisulfite-treated DNA was obtained using an EZ DNA Methylation-Lightning Kit (Zymo Research Corp., Irvine, CA, USA). After bisulfite conversion, the *IGF1* P2 promoter was amplified by PCR as described previously [18]. Methylation analyses of CG108, CG137, CG207, CG218, CG224, and CG232 (chr12: 102873145-102873269 in GRCh37/hg19) were performed (Fig. 1). Briefly, a 5 μL aliquot of the resulting 10 μL bisulfite-treated genomic DNA solution was subjected to polymerase chain reaction analysis to amplify the *IGF1* P2 promoter (hg18: chr12: 101396974-101397934), using the following primers and thermal conditions: Forward, 5′-AAT TTG GTT GTT GTT GTT AGT GTA T-3′, Reverse, 5′-AAT TAA ACC CTC AAA CAA TTA AAT C-3′; 95 °C for 10 min, [95 °C, 30 s; 54 °C, 1 min; 73 °C, 1 min] × 45 cycles, 73 °C for 5 min. TaKaRa Epi Taq^TM^ HS (Takara Bio Inc., Shiga, Japan) was used for amplification. The 175-bp amplicons were purified using gel-based clean up by MinElute Gel Extraction Kit (Qiagen). The purified amplicons were subjected to a NEBNext Ultra II DNA Library Prep Kit for Illumina (New England Biolabs Japan, Inc., Tokyo, Japan), including dual indexing. These libraries were multiplexed and sequenced on an Illumina MiSeq system (Illumina Inc., San Diego, CA, USA). The reads were aligned to an in silico converted reference using Bowtie2, and variant calling was used to identify the percentage of methylated cytosines using the Bicycle software [20]. The depth of sequencing reads ranged from 140–14682.

### Statistics

The results were analyzed by chi-square test, Mann–Whitney *U* test, and Spearman’s correlation analysis. Hierarchical cluster analysis of individual methylation patterns of six CpG sites on each haplotype was performed. All statistical analyses were performed using SPSS Statistics, version 24∙0 (IBM Corp., Armonk, NY, USA). In all analyses, *p* < 0.05 was considered to indicate statistical significance. The *z* scores of birth weight and length were calculated by subtracting the mean from the raw score, then dividing the difference by the population standard deviation using the Japanese datasets (http//jpse.umin.jp/taikakubirthlongcross1.xlsx).

## Results

### Participant characteristics

The backgrounds of participants are shown in Table 1. All participants were Japanese. The gestational age ranged from 25 weeks + 1 day to 31 weeks + 6 days among infants in the IUGR (29.4 [28.0, 30.7] weeks, median [25%, 75%], here and below), and from 23 weeks + 5 days to 31 weeks + 5 days among infants in the non-IUGR group (29.3 [26.0, 31.1] weeks). These ages did not significantly differ between groups (*p* = 0.869). The body weight ranged from 342 to 1006 g among infants in the IUGR group (720 [547, 987] g), and from 696 to 1900 g among infants in the non-IUGR group (1226 [858, 1496] g). Body weight at birth is significantly lower among infants in the IUGR group (*p* < 0.001). The body height ranged from 25.8 to 37.7 cm among infants in the IUGR group (31.1 [29.1, 35.8] cm), and from 27.0 to 44.0 cm among infants in the non-IUGR group (37.0 [33.8, 39.0] cm). Body height at birth was significantly shorter among infants in the IUGR group (*p* = 0.003). Fourteen of 19 infants in the IUGR group and 24 of 37 infants in the non-IUGR group were boys; there was no significant difference in sex distribution between groups. Regarding maternal contribution to intrauterine growth, the following factors were analyzed: maternal information; frequency of pregnancy by in vitro fertilization (IVF), frequency of twin pregnancy (twin), and frequency of hypertensive disorders of pregnancy (HDP) diagnosed by an obstetrician. HDP was diagnosed by hypertension (systolic BP ≥ 140 and/or diastolic BP ≥ 90 mmHg) and proteinuria (spot urine protein/creatinine ratio ≥ 30 mg/mmol). Among infants in the IUGR group, one IVF, one twin, and nine HDP were observed. Among infants in the non-IUGR group, six IVF, 10 twin, and three HDP were observed. There were no significant differences in frequency of IVF and twin pregnancies *(p* = 0.241, *p* = 0.202, respectively). Nine of the 19 infants in the IUGR group had HDP, which was significantly higher than the rate in the non-IUGR infant group (*p* = 0.001).

**Table 1:** Patient characteristics

	IUGR (*n* = 19)	Non-IUGR (*n* = 37)	*P* value
Gestational age (weeks), median [25%, 75%]	29.4 [28.0, 30.7]	29.3 [26.0, 31.1]	0.869
Body weight (g), median [25%, 75%]	720 [547, 987]	1226 [858, 1496]	< 0.001
Body height (cm), median [25%, 75%]	31.1 [29.1, 35.8]	37.0 [33.8, 39.0]	0.003
Sex (male, *n*)	14	24	0.290
In vitro fertilization (*n*)	1	6	0.241
Hypertensive disorders of pregnancy (*n*)	9	3	0.001
Twin pregnancy (*n*)	1	10	0.202
Broncho pulmonary dysplasia (*n*)	12	18	0.086
Abnormal MRI findings(*n*)	1	10	0.241
Retinopathy of prematurity (*n*)	2	5	0.749

The differences between groups were analyzed by Mann-Whitney *U* test or chi-square test

To assess the postnatal influence of IGF1 secretion, we investigated the frequency of infantile complications that may be associated with low IGF1 concentration, such as bronchopulmonary dysplasia (BPD; defined as the need for additional oxygen after the age of 28 days), retinopathy of prematurity (ROP; defined as the need for treatment by an ophthalmologist), and poor brain development (defined as abnormal brain magnetic resonance imaging findings (MRI) at the time of discharge). BPD was observed in 12 infants in the IUGR group and 18 infants in the non-IUGR group. ROP was observed in two infants in the IUGR group and five infants in the non-IUGR group. Abnormal MRI findings were observed in one infant in the IUGR group and 10 infants in the non-IUGR group. There was no difference between the two groups with regard to clinical complications during the neonatal period (BPD *p* = 0.086, ROP *p* = 0.749, MRI *p* = 0.241).

### Methylation analysis of *IGF1* P2 promoter

**Fig. 2:**
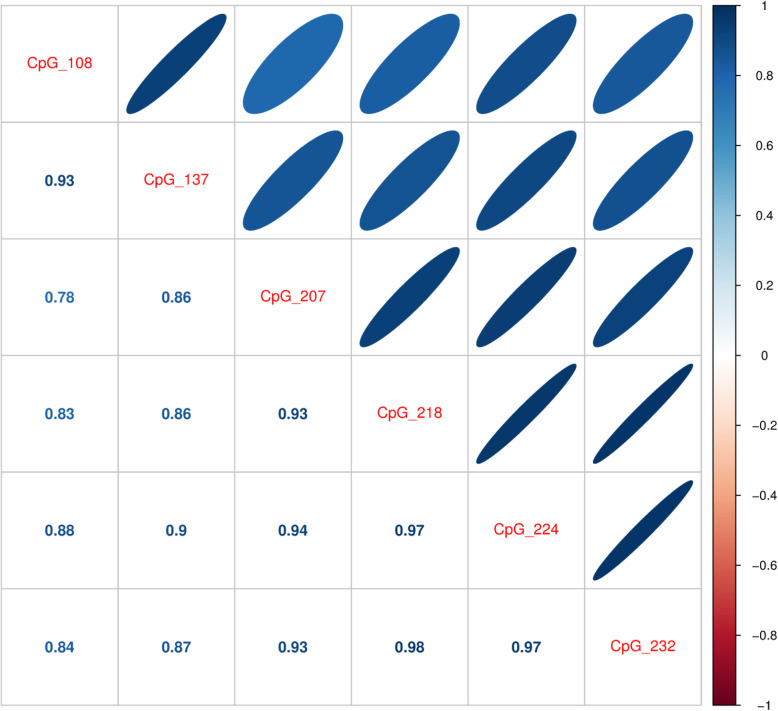
Correlation of methylation status between the six CG sites. Correlation coefficients were calculated by Spearman’s correlation analysis. All correlations were significant (*p* < 0.001)

**Fig. 3:**
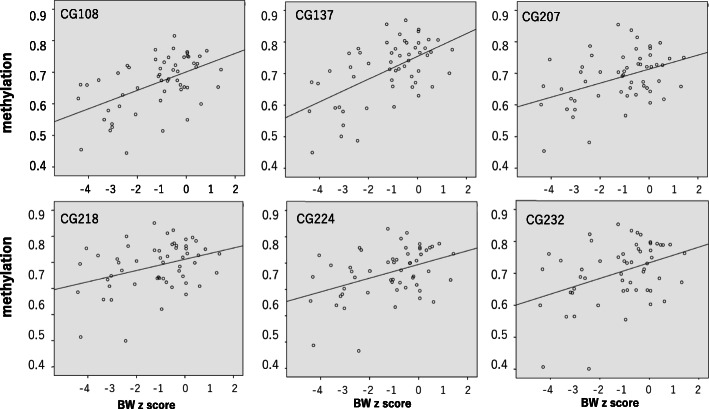
Correlation between methylation of six CpG sites and birth weight. Methylation of six CpG sites showed positive correlations with birth weight (CG108: *r* = 0.506, *p* < 0.001; CG137: *r* = 0.547, *p* < 0.001; CG207: *r* = 0.354, *p* = 0.007; CG218: *r* = 0.310, *p* = 0.020; CG224: *r* = 0.373, *p* = 0.005; CG232: *r* = 0.348, *p* = 0.009) analyzed by Spearman’s correlation analysis

**Fig. 4:**
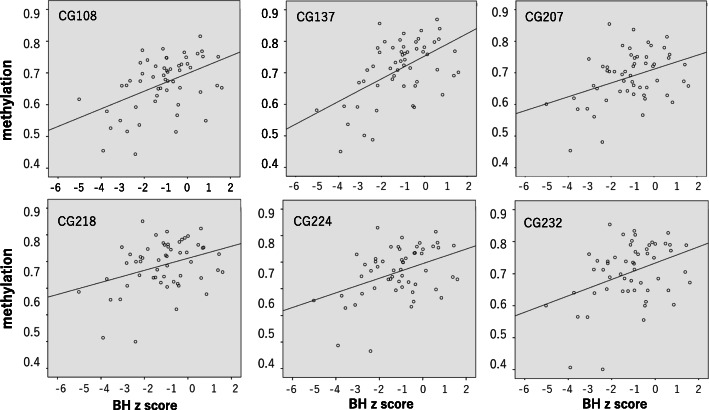
Correlation between methylation of six CpG sites and birth length. Methylation of six CpG sites showed positive correlations with birth length (CG108: *r* = 0.453, *p* < 0.001; CG137: *r* = 0.515, *p* < 0.001; CG207: *r* = 0.333, *p* = 0.012; CG218: *r* = 0.311, *p* = 0.020; CG224: *r* = 0.375, *p* = 0.004; CG232: *r* = 0.346, *p* = 0.009) analyzed by Spearman’s correlation analysis

**Fig. 5:**
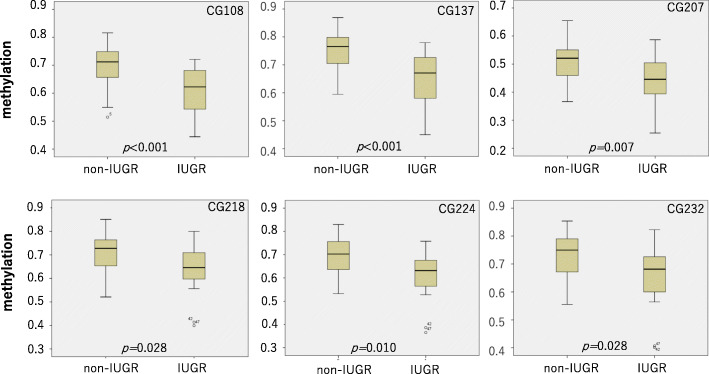
Methylation of six CpG sites of infants in the IUGR and non-IUGR groups. Methylation was significantly lower in the IUGR group than in the non-IUGR group in all six CG sites (CG108, 0.63 [0.54, 0.69] vs 0.71 [0.65, 0.75], *p* < 0∙001; CG137, 0.67 [0.58, 0.73] vs 0.77 [0.70, 0.80], *p* < 0.001; CG207, 0.45 [0.39, 0.51] vs 0.52 [0.46, 0.55], *p* = 0.012; CG218, 0.65 [0.60, 0.72] vs 0.73 [0.65, 0.76], *p* = 0.028; CG224, 0.64 [0.56, 0.69] vs 0.70 [0.64, 0.76], *p* = 0.010; CG232, 0.69 [0.60, 0.74] vs 0.75 [0.67, 0.79], *p* = 0.028, median [25%, 75%], IUGR group vs non-IUGR group, respectively)

**Fig. 6:**
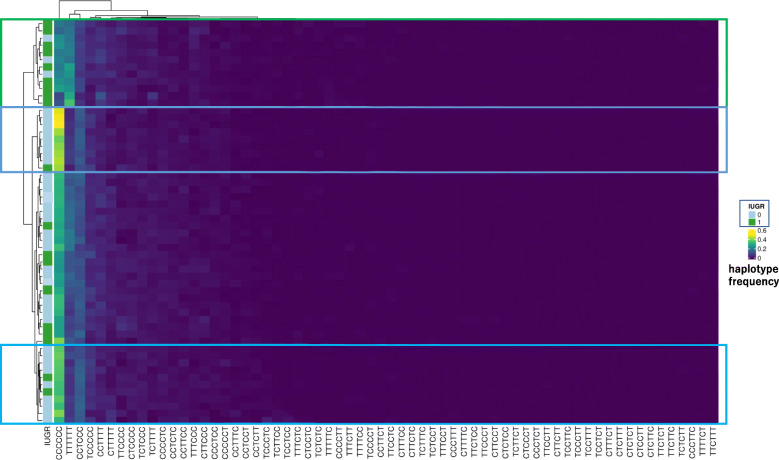
Haplotype methylation analysis of individuals. C represents a methylated CpG site, and T represents an unmethylated CpG site. CCCCCC means CG107, CG137, CG207, CG218, CG224, and CG232 were methylated, respectively. The frequency of each haplotype was shown as a heatmap. Hierarchical cluster analysis revealed a cluster surrounded by green where IUGR infants were dominant and relatively high TTTTTT and low CCCCCC were seen. There were two clusters surrounded by blue where non-IUGR infants were dominant and relatively high CCCCCC and low TTTTTT were seen

The results of methylation analysis are shown in Table 2 and Figs. 2, 3, 4, 5, and 6. The methylation states of CG108, CG137, CG207, CG218, CG224, and CG232 were highly correlated (range, 0.861–0.983, Fig. 2). The correlation coefficients of CG108 with other CG sites were 0.93, 0.78, 0.83, 0.88, and 0.84 (vs CG137, CG207, CG218, CG224, and CG232, respectively). CG137 correlation coefficients were 0.86, 0.86, 0.90, and 0.87 (vs CG207, CG218, CG224, and CG232, respectively). CG207 correlation coefficients were 0.93, 0.94, and 0.93 (vs CG218, CG224, and CG232, respectively). Those of CG218 were 0.97 and 0.98 (vs CG224 and CG232, respectively), and CG224 vs CG232 was 0.97. All these results were statistically significant (*p* < 0.001). The methylation of six CpG sites positively correlated with body weight (CG108: *r* = 0.506, *p* < 0.001; CG137: *r* = 0.547, *p* < 0.001; CG207: *r* = 0.354, *p* = 0.007; CG218: *r* = 0.310, *p* = 0.020; CG224: *r* = 0.373, *p* = 0.005; CG232: *r* = 0.348, *p* = 0.009) (Fig. 3) and body length at birth (CG108: *r* = 0.453, *p* < 0.001; CG137: *r* = 0.515, *p* < 0.001; CG207: *r* = 0.333, *p* = 0.012; CG218: *r* = 0.311, *p* = 0.020; CG224: *r* = 0.375, *p* = 0.004; CG232: *r* = 0.346, *p* = 0.009) (Fig. 4). As shown in Fig. 5, methylation was significantly lower at all six CG sites in the IUGR group compared with the non-IUGR group (CG108, 0.63 [0.54, 0.69] vs 0.71 [0.65, 0.75], *p* < 0.001; CG137, 0.67 [0.58, 0.73] vs 0.77 [0.70, 0.80], *p* < 0.001; CG207, 0.45 [0.39, 0.51] vs 0.52 [0.46, 0.55], *p* = 0.012; CG218, 0.65 [0.60, 0.72] vs 0.73 [0.65, 0.76], *p* = 0.028; CG224, 0.64 [0.56, 0.69] vs 0.70 [0.64, 0.76], *p* = 0.010; CG232, 0.69 [0.60, 0.74] vs 0.75 [0.67, 0.79], *p* = 0.028, median: [25%, 75%], IUGR group vs non-IUGR group, respectively).

**Table 2:** Subgroup analysis of methylation with and without IUGR and HDP

		CG108	CG137	CG207	CG218	CG224	CG232
HDP **(−)** median		0.70	0.75	0.51	0.71	0.68	0.72
	25%	0.64	0.67	0.44	0.63	0.61	0.65
	75%	0.74	0.78	0.55	0.76	0.75	0.79
HDP **(+)** median		0.65	0.69	0.46	0.69	0.65	0.71
	25%	0.57	0.59	0.42	0.63	0.58	0.62
	75%	0.70	0.76	0.53	0.75	0.70	0.76
*p* value		0.13	0.17	0.27	0.76	0.33	0.61
IUGR **(−)** HDP(-) median		0.71	0.77	0.52	0.72	0.71	0.74
	25%	0.65	0.70	0.46	0.65	0.64	0.67
	75%	0.75	0.80	0.55	0.76	0.76	0.79
IUGR **(−) ** HDP(+) median		0.71	0.77	0.53	0.75	0.70	0.76
	25%	0.55	0.63	0.40	0.58	0.57	0.60
	75%	0.76	0.84	0.63	0.83	0.80	0.83
*p* value		0.98	0.86	0.90	0.60	0.90	0.82
IUGR **(+) ** HDP(-) median		0.59	0.67	0.44	0.64	0.63	0.68
	25%	0.52	0.50	0.36	0.56	0.53	0.56
	75%	0.69	0.74	0.52	0.71	0.68	0.71
IUGR **(+)** HDP(+) median		0.63	0.67	0.45	0.67	0.65	0.69
	25%	0.57	0.59	0.42	0.63	0.58	0.62
	75%	0.67	0.72	0.49	0.72	0.68	0.74
*p* value		0.86	1.00	0.86	0.55	0.70	0.60

The methylation of each CpG site in infant subgroups is shown as median, 25%, 75%. *p* was calculated by Mann-Whitney *U* test

HDP: Hypertensive disorders of pregnancy

To assess the influence of maternal HDP on CG methylation, we performed subgroup analysis with and without maternal HDP and IUGR. As shown in Table 2, there was no significant difference in methylation of all six CGs in IUGR, non-IUGR, and whole group infants.

Figure 6 shows the methylation haplotype pattern of each individual. CCCCCC represents the methylated haplotype at CG108, CG137, CG207, CG218, CG224, and CG232, respectively. However, TTTTTT means that all 6 sites were unmethylated. Hierarchical cluster analysis revealed a cluster surrounded by green where IUGR infants were dominant and relatively high TTTTTT and low CCCCCC were seen. There were 2 clusters surrounded by blue where non-IUGR infants were dominant and relatively high CCCCCC and low TTTTTT were seen.

## Discussion

In contrast to term infants and children, *IGF1* P2 promoter methylation levels were significantly reduced in infants with IUGR. Our findings suggested that preterm infants can adapt to intrauterine adverse nutritional environments via epigenetic modification of DNA in the early developmental stages, and that > 32 weeks of gestation is the critical time window for programming of later body growth through *IGF1* gene methylation.

Children’s heights are known to be highly correlated with their parents’ heights [11]. Therefore, programming of growth-related genes, including *IGF1*, may occur at least in part in the early embryonic stage, such as the postimplantation stage. The results of the present study showed that IGF1 secretion is epigenetically regulated to be high in IUGR infants born at < 32 weeks of gestation, in contrast to term newborn infants and children. This finding indicated that methylation of the *IGF1* gene has the ability to respond to in utero malnutrition before 32 weeks of gestation and may be reset at 32–37 weeks of gestation. Epigenetic reprogramming has been demonstrated in mammals at distinct, key developmental stages, such as in the zygote, in the primordial germ cells, and during early body development. There have been a few reports that epigenetic reprogramming occurs after organogenesis, such as glucocorticoid receptor gene methylation by the postnatal environment in preterm infants [21].

HDPs are associated with low birth weight and represent a major cause of maternal and/or perinatal mortality and morbidity. The mechanisms underlying HDP-associated complications are poorly understood, but epigenetic patterns contribute to the pathogenesis of fetal complications. Epigenome-wide DNA methylation analysis in cord blood of term infants revealed that HDP were associated with DNA methylation at 43 CpG sites [22]. The *IGF1* P2 promoter region was not covered by the analysis. Thus, it is unclear whether it was differentially methylated in infants with maternal HDP. The present study indicates that maternal HDP may not have a large effect on *IGF1* P2 promoter methylation.

Haplotype methylation analysis revealed that methylation change tended to occur simultaneously at several CpG sites. It seems to be important for future study.

A limitation of the present study was its lack of data regarding serum IGF1 concentrations. This was due to the large sample volume required from infants with a low body weight of 342–1900 g and the risk of postnatal hypovolemia. It is known that serum IGF1 concentrations have been positively correlated with birth weight and length in preterm infants [23]. Therefore, the IGF1 concentration may have been lower in the IUGR group than in the non-IUGR group in the present study due to intrauterine malnutrition status, despite epigenetic activation of the *IGF1* gene.

After preterm birth, there is a decline in serum IGF1 concentration that is induced by undernutrition, deficient nutrition utilization [24], hypoxia [25], inflammation [26], and genetic factors, as well as hormones (e.g., thyroid hormone and cortisol). A prolonged duration of low serum IGF1 in extremely premature infants is strongly associated with increased risks of multiple major neonatal morbidities, which have a significant impact on long-term health [27–29], including BPD, ROP, and poor brain development. There were no differences in rates of these complications between the two groups in the present study. Therefore, the effect of low methylation of the *IGF1* gene may have been reduced by severe conditions to produce IGF1 after preterm birth.

Tissue differences in epigenomic status represent the most important limitation of epigenetic studies in human subjects, including the present study. However, the tissue differences in *IGF1* gene methylation are relatively well characterized. It has been shown that the methylation levels were comparable in white blood cells, peripheral blood mononuclear cells, and CD4 T lymphocytes. The methylation levels were approximately 50% lower for most P2 CGs in the liver and growth plates, which are the main sites of IGF1 production [17]. Therefore, the use of white blood samples in the present study was presumed to cause few problems related to tissue differences.

The evaluation of IGF1 gene methylation after 32 weeks of gestation in the uterus or after preterm birth may be important for perinatal management of infants with IUGR. Further, longitudinal investigations are needed along with the development of methods for analyzing the fetal epigenome using cell-free DNA in maternal blood.

Studies examining the epigenetic status of the *IGF1* gene in deregulated lipid metabolism, cardiovascular disease, and diabetes are limited. *IGF1* P1 promoter methylation levels were increased in type 2 diabetes patients compared with normal glucose tolerance subjects, while serum IGF1 levels were lower in type 2 diabetes [30]. Additional epigenetic studies of *IGF1* P2 promoter methylation dynamics are needed to understand these interactions further.

## Conclusions

It has been suggested that > 32 weeks of gestation may be the critical time window for programming of later body growth through *IGF1* gene methylation.

This study may provide new insights to prevent the onset of adult diseases and to aid in nutritional management for preterm birth infants in neonatal intensive care units.

## Data Availability

The datasets used and/or analyzed during the current study are available from the corresponding author on reasonable request.

## Abbreviations

IGF1: Insulin-like growth factor 1
IUGR: Intrauterine growth restriction
IVF: In vitro fertilization
HDP: Hypertensive disorders of pregnancy
BPD: Bronchopulmonary dysplasia
ROP: Retinopathy of prematurity
